# Investigation of Social Constraints, Psychosocial Adjustment and Optimism among Dialysis Patients

**DOI:** 10.3390/clinpract14040115

**Published:** 2024-07-19

**Authors:** Eirini Zorba, Georgia Fasoi, Eirini Grapsa, Afroditi Zartaloudi, Maria Polikandrioti, Victoria Alikari, Areti Stavropoulou, Chrysoula Dafogianni, Orchan Impis, Georgia Gerogianni

**Affiliations:** 1 Department of Nursing, University of West Attica, 12243 Athens, Greece; ezormpa@uniwa.gr (E.Z.); gfasoi@uniwa.gr (G.F.); azarta@uniwa.gr (A.Z.); mpolyk@uniwa.gr (M.P.); vicalikari@uniwa.gr (V.A.); astavropoulou@uniwa.gr (A.S.); cdafog@uniwa.gr (C.D.); oimpis@uniwa.gr (O.I.); 2 Department of Nephrology, Aretaieio Hospital, National and Kapodistrian University of Athens, 11528 Athens, Greece; egrapsa@aretaieio.uoa.gr

**Keywords:** social constraints, psychosocial adjustment, optimism, dialysis patients

## Abstract

Background: Social constraints are perceived as unsupportive behaviors, leading to inadequate psychosocial adjustment, while optimism can help people recover from distress and reduce any negative effects of chronic disease. The aim of this study was to investigate social constraints, psychosocial adjustment and optimism among patients on dialysis. Methods: In this study, 402 patients undergoing dialysis in Greece completed the following questionnaires: (i) the Social Constraints Scale (SCS) for the assessment of social constrains, (ii) the Psychosocial adjustment to illness scale (PAIS-SR) for the assessment of psychosocial adjustment, and (iii) the LOT-R scale for the assessment of optimism. A Mann–Whitney test was used for the comparison of continuous variables between two groups. Spearman correlation coefficients (rho) were used to explore the association of two continuous variables. Multiple linear regression analysis was used with the SCS scale. Results: Greater difficulty in psychosocial adjustment in the domestic, vocational, extended family and social environments, sexual relationships, and health care as well as greater psychological distress were significantly associated with a greater occurrence of social constraints (*p* < 0.001). Additionally, greater optimism was significantly associated with fewer social constraints and lower difficulty in adjusting to their disease (*p* < 0.001). Conclusions: Greater difficulty in all dimensions of psychosocial adjustment is associated with more social constraints, while optimism is associated with fewer social constraints and better disease adjustment.

## 1. Introduction

Social constraints are caused by a socially non-susceptible environment which prevents thought discussion associated with stress and feelings, leading to avoidance. People who experience a trauma perceive social constraints as unsupportive behaviors, meaning that there are no people available to listen to or talk to them concerning the problematic situation, recommendations to people who experience trauma to cover their emotions and worries, pretending or distracting from the problem, as well as critical verbal expressions. These social constraints frequently lead to psychological distress among people who experience a trauma [1].

The initiation of dialysis therapy is a traumatic experience for patients since they face serious physical, psychological, and social problems [2] due to limitations caused by the treatment [3]. More specifically, patients have dietary and fluid restrictions, difficulty with vacations, long time spent on dialysis, fatigue, insufficient sexual activity, frequent hospital admissions, financial difficulties, uncertainty about the future, limited social life, and changes in family roles [4]. They also suffer from body image disorders [5], reduced functionality and loss of roles related to work [6].

Additionally, dialysis patients have a variety of comorbidities, such as cardiovascular diseases, malnutrition, cognitive decline, chronic pain, and poor sleep quality [7]. Freedom and independence loss is the most difficult part of their existence. Their sickness leads them to have a feelings of powerlessness, despair and being a burden to others, asking for help in order to endure their routine [8]. Thus, they frequently have depressive disorders and experience feelings of fear, anxiety, and hopelessness [7].

The focus on psychosocial adaptation is about accepting the dialysis necessity. It includes a procedure where individuals can move through a situation of rejecting to accepting and from disengaging to engaging [2]. Adaptation is an ongoing procedure, and in most chronic illnesses, there are always new instructions that people must adapt to. Thus, the concept of adaptation as a procedure can be helpful in trying to help individuals adapt more effectively to their treatment [9]. Proper adaptation perhaps includes a sense of control over the disease management, acceptance of the disease, and high social support, in combination with coping strategies focused on problem, positive health behaviors, and compliance with treatment [10]. However, poor adjustment to dialysis therapy usually leads to non-compliance to therapy, depressive disorders, inefficient coping, low social functioning, and poor quality of life. Difficulties in adjustment are usual in the dialysis population and can have a negative impact on treatment and specifically on non-compliance [9].

Being optimistic, the tendency to expect a favorable future, can be an efficient approach for coping with environmental stressors. People with optimism usually have increased physical and psychological well-being [11]. Thus, optimism has a positive impact on health, since it can help people recover from distress, maintain resilience, and reduce any negative effects of chronic disease [12], while it leads to efficient problem solving and increased health perception [11]. The purpose of this study was the investigation of social constraints, psychosocial adjustment and optimism among patients on dialysis.

## 2. Materials and Methods

### 2.1. Study Sample

The study sample consisted of 402 patients undergoing dialysis treatment (72.1% hemodialysis and 27.9% peritoneal dialysis) in Greece. Being between 18 and 85 years of age, undergoing dialysis for at least three months, and having abilities of speech, reading, and writing in Greek were the inclusion criteria for participation in the study. Exclusion criteria were inadequate language abilities, age over 85 years old, cognitive impairment, and drug or alcohol abuse.

This study lasted from December 2022 to September 2023. Data collection was carried out by interviews using the following questionnaires: (i) Social Constraints Scale for the assessment of social constrains, (ii) Psychosocial adjustment to illness scale (PAIS-SR) for the assessment of psychosocial adjustment, (iii) Life Orientation Test for the assessment of optimism (LOT-R), and (iv) a questionnaire about demographic characteristics.

### 2.2. Instruments

#### 2.2.1. Social Constraint Scale (SCS)

The Social Constraint Scale (SCS) is a self-administered questionnaire which includes fifteen questions and investigates how frequently during the past month individuals’ significant others exhibited the behaviors described in the questions of the scale (e.g., “How frequently in the past month has your spouse/partner changed the subject when you tried to discuss your disease?”). Respondents could choose the answer from a 4-point Likert scale. The total score ranged from 15 to 60, where the higher the score, the greater the occurrence of social constraints. Factor analysis revealed the following three underlying factors in the Greek Social Constraints Scale: unsupportive behaviors, avoidant behaviors, and suggestions for pretense and distraction. The subscale reliability was satisfactory in the Greek population since Cronbach’s alpha ranged from 0.77 to 0.88. All subscales had a significant association with intrusions and psychological distress. Thus, the Greek Social Constraints Scale is a reliable and valid multidimensional questionnaire [1].

#### 2.2.2. Psychosocial Adjustment to Illness Scale (PAIS-SR)

The Psychosocial adjustment to illness scale (PAIS-SR) reflects adjustment in seven significant psychosocial domains: (a) the health care orientation evaluates patients’ attitudes on health matters, (b) the vocational environment evaluates the effect of the disease on patients’ vocational issues, (c) the domestic environment evaluates participants’ level of adjustment to family issues, including financial effect of the disease, quality of relations, and family communication, (d) the sexual relationships estimates the effect of the disease on patients’ sexual well-being, (e) the extended family relationships estimates the possible difficulties of patients with extended family members, (f) the social environment investigates patients’ adaptation in social activities, and (g) the psychological distress examines patients’ psychological discomfort due to an illness.

For each of the 46 questions, participants were asked to choose on a 4-point Likert scale (0–3) the answer that best described the impact of their disease on their life in the past 30 days. Higher scores showed a lower level of adaptation. The Greek version of the PAIS-SR has acceptable internal consistency reliability (Cronbach’s alpha coefficients > 0.62) and construct validity as well as satisfactory convergent validity [13].

#### 2.2.3. LOT-R Scale

The LOT-R assesses dispositional optimism and includes ten questions on a scale of 0–4 that measure optimism. Higher scores indicate higher levels of optimism. The LOT-R has good internal consistency (Cronbach’s alpha = 0.71) and good convergent validity in the Greek population. Thus, it appears to be a valid tool in the assessment of dispositional optimism in Greek people [14].

### 2.3. Statistical Analysis

Quantitative variables were expressed as mean values (standard deviation) and as median (interquantile range), while categorical variables were expressed as absolute and relative frequencies. A Mann–Whitney test was utilized for the comparison of continuous variables between two groups. Spearman correlations coefficients (rho) were utilized to explore the association of two continuous variables. Multiple linear regression analysis was used with the SCS scale. The regression equation included terms for participants’ demographical and clinical characteristics, LOT-R scale and the PAIS-SR subscales. PAIS-SR subscales were entered one at a time in the analysis due to being highly correlated with each other. Adjusted regression coefficients (β) with standard errors (SE) were computed from the results of the linear regression analyses. For the investigation of the mediating role of the LOT-R scale in the association between the PAIS-SR and SCS scales, SPSS PROCESS macro as well as the analysis strategy of Baron and Kenny was used following Hayes’ guidelines [15].

A 1000-sample bootstrap procedure was used to estimate bias-corrected 95% confidence intervals (CIs) to test the significance of the indirect effect of the relationships. Mediation is presented when the indirect effect is significant, i.e., if confidence intervals do not contain zero. According to Hayes [16] and colleagues [17,18], this bootstrapping procedure overcomes the limitations of the approaches highlighted by Baron et al. [19] and Sobel [20], yielding results that are more accurate and less affected by sample size. Full mediation is presented when the direct effect is not significant, while partial mediation is presented when the direct effect is significant. All reported p-values are two-tailed. Statistical significance was set at *p* < 0.05, and analyses were conducted using SPSS statistical software (version 26.0).

### 2.4. Ethics

Before collecting data, we obtained approval from the Ethics Committee of the University of West Attica (approval: 42/18-11-2022, approval date: 18 November 2022). All participants were informed during their dialysis therapy of the purpose and procedure of the study and the anonymity of the data. The study was carried out in accordance with the Declaration of Helsinki (1989).

## 3. Results

### 3.1. Participants’ Characteristics

A total of 402 participants were participated in the study. Their mean age was 62 years (SD = 13.8 years), and most of them were males (65.7%). The majority of the sample were Greeks (93.5%), married (60.7%), high school graduates (25.9%), with medium economic status (67.9%). Most of the participants were on hemodialysis (72.1%), and the remaining were on peritoneal dialysis (27.9%) (Table 1).

### 3.2. Correlation of Social Constraint (SCS), Psychosocial Adjustment to Illness (PAIS-SR), and LOT-R Optimism Scales

All the SCS subscales were significantly and positively correlated with all the Psychosocial adjustment to illness subscales (PAIS-SR). Thus, greater difficulty in psychosocial adjustment in the domestic, vocational, extended family and social environment, sexual relationships, and health care as well as greater psychological distress were significantly associated with a greater occurrence of social constraints (unsupportive behaviors, avoidant behaviors, suggestions for distraction and pretense) (*p* < 0.001). On the contrary, greater optimism was significantly associated with fewer social constraints and lower difficulty in adjusting to their disease (*p* < 0.001).

Spearman correlation coefficients (rho) among SCS, PAIS-SR and LOT-R are shown in Table 2.

### 3.3. Effect of Total Social Constraint Scale (SCS) on Psychosocial Adjustment to Illness (PAIS-SR), and LOT-R Optimism Scale

After adjusting for demographical and clinical characteristics, the total Social Constraint Scale (SCS) score continued to be significantly associated with the PAIS-SR and LOT-R scales, which was similar to the univariate method (Table 3). Thus, greater difficulty in adjusting to the disease was associated with more frequent social constraints (<0.001), while increased optimism was associated with fewer social constraints (less occurrences of non-supportive behaviors, avoidant behaviors, suggestions for distraction and pretense) (<0.001) and less difficulty adjusting in the domestic sphere, vocational environment, sexual relationships, extended family relationships, social environment, and health care as well as lower psychological distress (<0.001).

## 4. Discussion

The present study found that greater difficulty in psychosocial adjustment in the domestic and extended family environment and greater psychological distress were significantly associated with a greater occurrence of social constraints (unsupportive behaviors, avoidant behaviors, suggestions for distraction, and pretense). This can be viewed in the context of disrupted family relationships, as family caregivers frequently feel disappointed due to the responsibilities of dialysis treatment and the provision of prolonged care, resulting in physical and psychological burden, social isolation and difficulties in marital relationships [4,21]. In a similar study, it was found that women who provided care to their husbands had high anxiety levels due to their responsibilities for the care of their children and their family at the same time [4]. Additionally, patients on dialysis frequently change roles in the family, which increases their psychological distress [6]. Thus, spouses often feel anger toward their patients when there are role conflicts in the family and especially when patients are unable to perform certain tasks and are not providing a living [6].

Moreover, in this study, we found that greater difficulty in the social environment was significantly associated with a greater occurrence of social constraints. This can be attributed to the fact that patients on dialysis usually have a limited social life, which leads to social isolation [4]. Loneliness is related to a loss of solidarity, resulting in isolation [22]. Thus, patients who are socially isolated are less likely to ask for help for health care and health education because of their small social environment. Having a long-term disease, limited social environment and low health education can lead to inefficient coping, feelings of loneliness, poor adjustment, and psychological distress [23]. In a similar study, high anxiety and depression levels had a close association with the low social support experienced by dialysis patients, which was possibly due to emotional disorders and limited social relations [24].

In addition, the results of this study indicated that greater difficulty in the vocational environment was significantly associated with a greater occurrence of social constraints. Similarly, Balley et al. [5] found that young individuals on dialysis had limited career prospects due to their illness, leading to unpleasant jobs offered compared to the ones they wanted. Thus, they had a sense of uncertainty and social isolation [5]. It is important to take into consideration that more than 75% of patients on hemodialysis are unable to have a full-time job after the initiation of their treatment [25]. This can be attributed to the fact that dialysis people frequently have comorbidities, hospital admissions, and fatigue that make them unable to maintain their work [7].

The present study also found that greater difficulty in sexual relationships was significantly associated with a greater occurrence of social constraints. Similarly, it was found that patients on dialysis who had a fistula or catheter reported that their sex life was negatively affected and it limited their positions during sexual activity. Additionally, they did not desire sexual activity because of the dialysis catheter for fear of it becoming wet, disconnected, infected, or damaged. Dialysis people who experienced sexual issues had low self-esteem, feelings of inadequacy, and a negative body image [26]. However, Moore et al. [27] found that in the early stages of dialysis, partners carried the burden and both parties experienced changes in their identities. Partners who had a good relationship with each other corrected the negative effects of early hemodialysis through positivity or acceptance of hemodialysis.

Additionally, the results of this study showed that greater optimism was significantly associated with fewer occurrences of non-supportive behaviors. Hope is a multidimensional concept that can help patients overcome difficulties in their life. Hope can alter the way patients on dialysis experience chronic kidney disease and their perspective on life. It is important to take into consideration that the behaviors and attitudes of health-care professionals can influence dialysis patients’ views of hope [6]. Thus, in the daily clinical setting, the measurement of social constraints and psychosocial adjustment should be an integral part of care, since early screening is helpful to provide individualized care according to patients’ difficulties.

Moreover, this study showed that increased optimism was significantly associated with less difficulty in psychosocial adjustment. This can be attributed to the fact that people with positive thoughts cope effectively with psychological stress and create a supportive environment by using efficient methods, such as reappraisal and problem solving [28]. Similarly, it has been found that people who have an internal desire to achieve personal purposes and expect the best possible outcomes can have a long healthy life. Thus, it can be assumed that maintaining or enhancing optimism and hope is related to the alleviation of distress and maintaining resilience [12]. It is important to take into consideration that hope and optimism have a positive effect on health, leading to a reduction in the negative impact of chronic illness [12].

Additionally, optimism has been associated with increased cardiovascular health, low levels of stress, and low levels of inflammation. Optimistic people usually have adequate self-regulation, since they increase personal attempts when circumstances are positive and decrease attempts when circumstances are negative [11]. Therefore, efficient interventions for improving hope in dialysis people include counseling, social support from family, friends and caregivers, spiritual therapy, network support groups and stress management [29,30].

The findings of this study provide important information to health professionals about social constraints, psychosocial adjustment and optimism in the dialysis population. The results of the present study showed that greater difficulty in psychosocial adjustment was significantly associated with a greater occurrence of social constraints, while increased optimism was significantly associated with fewer social constraints and less difficulty in psychosocial adjustment.

It is important to take into consideration that dialysis patients must continue psychosocial adjustment during their illness, which frequently results in low psychological well-being [31]. Thus, social support from family and health professionals can have a protective role in the psychological status in these patients [32], while access to information and support services for couples before the initiation of dialysis can reduce the negative impact of dialysis on their relationships [27]. An increased level of hope among the dialysis population decreases their anxiety, worries, and sadness and improves the quality of their life [30].

However, the evaluation of other parameters, such as anxiety and depression, that may influence the level of social constraints, psychosocial adjustment and optimism in patients on dialysis was not the matter of enquiry in the present research. It is interesting to assess such issues in future research.

The present study was of cross-sectional design, and there was no evidence of causal relationship between parameters under evaluation. The method of convenience sampling is not representative of all dialysis patients living in Greece, thus limiting the generalizability of the results. Moreover, there was no next measurement in time that would allow the evaluation of possible changes in all dimensions under assessment.

## 5. Conclusions

The study highlights the significant impact of social constraints on the psychosocial adjustment of patients undergoing renal replacement therapy. Unsupportive and avoidant behaviors, along with suggestions for distraction and pretense, exacerbate difficulties in various aspects of patients’ lives, including domestic, vocational, extended family, social environments, sexual relationships, health care, and psychological well-being. Conversely, optimism emerges as a protective factor, positively influencing patients by reducing social constraints and facilitating better adjustment to their chronic condition. These results underscore the importance of fostering supportive social environments and enhancing optimism to improve psychosocial outcomes in this patient population. Efforts to mitigate social constraints and promote optimism could lead to better adherence to treatment and overall well-being.

## Figures and Tables

**Table 1:** Sample’s characteristics.

*n* = 402		*n* (%)
Sex	Male	264 (65.7)
	Female	138 (34.3)
Age, mean (SD)		62.0 (13.8)
Family status	Married	242 (60.7)
	Engaged	6 (1.5)
	In Relationship	16 (4)
	Widowed	53 (13.3)
	Divorced	26 (6.5)
	Single	56 (14)
Number of children	0	97 (24.1)
	1	67 (16.7)
	2	162 (40.3)
	3	51 (12.7)
	4	17 (4.2)
	5	6 (1.5)
	6	2 (0.5)
Educational level	Elementary	88 (21.9)
	Middle school	92 (22.9)
	High school	104 (25.9)
	University	92 (22.9)
	Master	20 (5)
	PhD	6 (1.5)
Economic status	Low	103 (25.6)
	Medium	273 (67.9)
	High	26 (6.5)
Greek Nationality	No	26 (6.5)
	Yes	376 (93.5)
Type of therapy	Hemodialysis	290 (72.1)
	Peritoneal dialysis	112 (27.9)
Duration (months), median (IQR)		35 (15–66)

**Table 2:** Spearman correlation coefficients (rho) among SCS, PAIS-SR and LOT-R optimism scale.

		Unsupportive Behaviors	Avoidant Behaviors	Suggestions for Distraction and Pretense	Social Constraint (SCS) Total	LOT-R Optimism Scale
Health Care Orientation	rho	0.28	0.24	0.17	0.26	−0.36
	***p***	**<0.001**	**<0.001**	**0.001**	**<0.001**	**<0.001**
Vocational Environment	rho	0.28	0.21	0.12	0.25	−0.32
	***p***	**<0.001**	**0.002**	0.079	**<0.001**	**<0.001**
Domestic Environment	rho	0.46	0.48	0.31	0.48	−0.42
	***p***	**<0.001**	**<0.001**	**<0.001**	**<0.001**	**<0.001**
Sexual Relationship	rho	0.24	0.30	0.16	0.26	−0.24
	***p***	**<0.001**	**<0.001**	**<0.001**	**<0.001**	**<0.001**
Extended Family Relationships	rho	0.40	0.44	0.24	0.42	−0.36
	***p***	**<0.001**	**<0.001**	**<0.001**	**<0.001**	**<0.001**
Social Environment	rho	0.33	0.35	0.22	0.34	−0.35
	***p***	**<0.001**	**<0.001**	**<0.001**	**<0.001**	**<0.001**
Psychological Distress	rho	−0.36	−0.31	−0.22	−0.35	0.39
	***p***	**<0.001**	**<0.001**	**<0.001**	**<0.001**	**<0.001**
PAIS-SR total	rho	0.45	0.42	0.20	0.42	−0.49
	***p***	**<0.001**	**<0.001**	**0.007**	**<0.001**	**<0.001**
LOT-R Optimism Scale	rho	−0.43	−0.39	−0.25	−0.41	−
	***p***	**<0.001**	**<0.001**	**<0.001**	**<0.001**	

**Table 3:** Multiple linear regression results having SCS total score as dependent variable.

	β+	SE++	b÷	*p*
Sex (Female vs. Male)	0.013	0.016	0.038	0.418
Married (Yes vs. No)	−0.004	0.016	−0.013	0.786
Age	0.001	0.001	0.015	0.780
Duration (months)	−0.001	0.001	−0.032	0.480
Type of therapy (Peritoneal dialysis vs. Hemodialysis)	−0.024	0.017	−0.066	0.153
Greek Nationality (Yes vs. No)	−0.062	0.032	−0.093	0.061
Educational level				
Elementary vs. Middle school	−0.004	0.023	−0.010	0.866
High school vs. Middle school	0.007	0.023	0.018	0.762
University vs. Middle school	−0.024	0.024	−0.060	0.324
Master/PhD vs. Middle school	−0.036	0.034	−0.054	0.301
Economic status				
Medium vs. Low	0.027	0.019	0.076	0.159
High vs. Low	0.057	0.035	0.085	0.107
LOT-R	−0.012	0.002	−0.319	**<0.001**
Health Care Orientation	0.008	0.002	0.197	**<0.001**
Vocational Environment	0.006	0.002	0.167	**0.016**
Domestic Environment	0.016	0.002	0.452	**<0.001**
Sexual Relationship	0.010	0.002	0.245	**<0.001**
Extended Family Relationships	0.018	0.002	0.370	**<0.001**
Social Environment	0.008	0.002	0.275	**<0.001**
Psychological Distress	0.011	0.002	0.346	**<0.001**

Note: Analysis was made with the logarithmic transformation of the dependent variable. PAIS-SR subscales were entered one at a time in the analysis due to being highly correlated with each other **β+** regression coefficient, **SE++** standard error, **b**÷ standardized regression coefficient.

## Data Availability

Data of the present study are not able to be shared due to issues of subjects’ privacy and confidentiality.
